# Integrating continuous differential evolution with discrete local search for meander line RFID antenna design

**DOI:** 10.1371/journal.pone.0223194

**Published:** 2019-10-21

**Authors:** James Montgomery, Marcus Randall, Andrew Lewis

**Affiliations:** 1 School of Technology, Environments and Design, University of Tasmania, Hobart, Tasmania, Australia; 2 Bond Business School, Bond University, Gold Coast, Australia; 3 School of Information and Communication Technology, Griffith University, Nathan, Australia; Northeast Electric Power University, CHINA

## Abstract

The automated design of meander line RFID antennas is a discrete self-avoiding walk (SAW) problem for which efficiency is to be maximized while resonant frequency is to be minimized. This work presents a novel exploration of how discrete local search may be incorporated into a continuous solver such as differential evolution (DE). A prior DE algorithm for this problem that incorporates an adaptive solution encoding and a bias favoring antennas with low resonant frequency is extended by the addition of the backbite local search operator and a variety of schemes for reintroducing modified designs into the DE population. The algorithm is extremely competitive with an existing ACO approach and the technique is transferable to other SAW problems and other continuous solvers. The findings indicate that careful reintegration of discrete local search results into the continuous population is necessary for effective performance.

## Introduction

Introduced in the middle of the last century [1], radio frequency identification (RFID) has become a near-ubiquitous way of tracking and identifying items in a variety of settings such as logistics and supply chains [2, 3]. An RFID system comprises two main components: a reader and a tag (that contains an antenna). The reader sends an RF signal that can power the receiver (the tag), which in turn radiates back a signal containing its ID to the reader [4]. This backscattered signal usually contains a number that uniquely identifies the tag, and hence item. A key design objective for the antenna is improving the read range (the distance the signal can be sent and received), which is generally inversely proportional to an antenna’s *resonant frequency*, *f*_0_, and proportional to its gain (related to its *efficiency*, *η*). Both these factors are determined by the design of the antenna, which consequently becomes a multiobjective optimization (MOO) problem with the objectives of minimizing *f*_0_ while maximizing *η*. Shorter antennas tend to be highly efficient, but resonate with frequencies far higher than those used in RFID systems. In contrast, longer antennas tend to have lower resonant frequencies, but are less efficient. The goal of this multiobjective problem is thus to produce an antenna with relatively low resonant frequency without sacrificing its efficiency. This is commonly achieved by producing antennas that (ideally) maximize the length of the antenna in a convoluted space-filling manner. While theoretical limits on antenna performance have been established [5] they are known to not be achievable in practice. Consequently, recent work by a number of authors [6–13] has used a variety of heuristic search techniques to explore the space of antenna designs and their associated performance, which is determined through simulation using a modern implementation of the NEC evaluation package [14].

**Fig 1:**
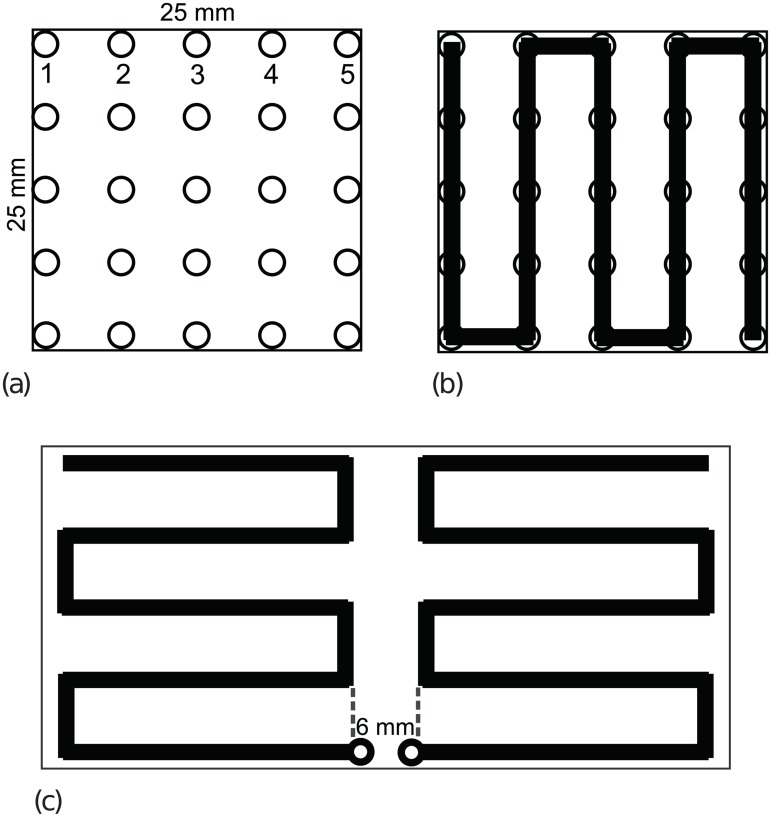
Antenna design space with example antenna. (a) A 5 × 5 antenna grid, with alternative start nodes labeled. (b) A meander line RFID antenna defined on that grid. (c) The complete dipole antenna produced by mirroring the solution in (b); excitation of the two halves occurs across the 6 mm gap.

Meander line RFID antennas are dipole antennas laid out on a Cartesian grid. Each side is the mirror image of the other, so only one half needs to be specified; the antenna is center-fed between these two halves (see Fig 1). The design of one half may start from any node along the edge facing the center. For a fixed physical design area the size of the grid can be varied to provide greater flexibility in possible designs and access to longer antenna designs. Each grid size is essentially a different, but related problem, with grids of 5×5 through 10×10 commonly used. The problem is closely related to that of designing self-avoiding walks (SAWs; see, e.g., Oberdorf et al. [15] or Sokal [16]). In the past, alternative designs have been manually produced by engineers such that the antenna’s path visits every node and hence is the maximum possible length. Recent work using heuristics for this problem has relaxed this constraint, ensuring that every solution is considered feasible while still allowing the solvers to produce high quality antennas [7–10, 13].

The first evolutionary algorithm (EA) applied to this problem was ant colony optimization (ACO; see, e.g., Dorigo and Stützle [17]), due to the natural alignment between ACO’s solution construction process and the process of constructing a SAW one segment at a time [11, 12]. While the the earliest applications solved the single objective of maximizing efficiency, later ACO approaches [10] treated the problem as multiobjective, introducing the commensurate goal of minimizing resonant frequency (the combination of low resonant frequency yet high efficiency is critical to maximizing the read range of the antenna). Using standard Pareto dominance based techniques [18] and a problem-specific local search operator known as *backbite*, Lewis et al. [10] found that low resonant frequency antennas could be produced while maintaining good efficiencies.

While ACO-based techniques display a natural suitability for the constructive design methods of meander line antennas, they are unlikely to be the only effective approaches to this problem. This is particularly true when the “problem” is to explore the performance envelope in the wider design space, not just optimise a specific antenna for a specific purpose. For these studies, using a diverse range of construction methods and optimisation algorithms is necessary to ensure a diversity of trial solutions and adequate coverage of the design space. To this end, later work investigated the efficacy of other, algorithmically-diverse techniques. Gomez-Meneses, Randall and Lewis [7] used another nature inspired technique referred to as extremal optimization (EO). As EO is an iterative, rather than constructive, heuristic, and handles permutations poorly, the antenna construction process was adapted to a knapsack problem in which antenna segments are selected for inclusion. This creates an extremely rich space of solutions compared to that explored by the ACO implementation, including antennas with “parasitic” elements that are not connected to the main antenna path. The results revealed that the EO algorithm had similar performance to ACO on the smaller grids (up to size 7), but dropped off, particularly in terms of resonant frequency, on larger grid sizes. Representing the problem as a binary selection problem is an ongoing area of research, with Hettenhausen, Lewis, Thiel and Shahpari [19] investigating the use of a binary Multi-Objective Particle Swarm Optimization algorithm (MOPSO) to explore the general design space of electrically-small, planar antennas using a representation of them as made up of a number of square patches. Preliminary experiments have shown some promising results in terms of the diversity of the design space explored but have, to date, not exceeded the performance envelope established by the original ACO studies.

To provide an additional contrasting approach to the ACO, the authors adapted the popular and widely-effective continuous solver differential evolution (DE; see, e.g., [20]) to this problem, with encouraging results [8], which were subsequently improved by biasing the algorithm’s exploration of the multiobjective search space towards regions of lower resonant frequency [9]. The DE employed a novel solution representation that encodes constructive decisions as real-valued numbers, and so operates in the space of meander line antennas rather than the larger solution space used by the EO. This early work demonstrated that the unusual choice of a continuous solution representation affords something that the intuitively well-matched ACO does not: the ability to recover from sequences of poor design decisions [21, 22]. However, the most common local search operator for SAWs, backbite, works directly on antenna designs and cannot be immediately applied to the real-valued vector solutions of DE. The present work investigates how best to integrate this discrete local search into the algorithm’s operation. The findings may support the application of other continuous algorithms to discrete problems.

The next section introduces the backbite operator [16] as a local search mechanism for meander line antenna design. This is followed by a description of the key algorithmic changes required to apply a continuous solver like DE to the discrete problem of RFID antenna design, and the particular design choices in the multiobjective DE algorithm used here. This includes the algorithm’s mechanism to bias its search towards antennas with lower resonant frequencies. The subsequent section describes how backbite local search is integrated into the DE-based solver, which is non-trivial as it works in the discrete space of antennas, so modified designs must then be mapped back into the continuous search space of DE. An empirical evaluation of combinations of the algorithm’s components—bias and local search—is then presented, followed by a summary of the implications of these findings for related problems and other continuous, population-based heuristics such as particle swarm optimization.

## Backbite local search for RFID antennas

**Fig 2:**
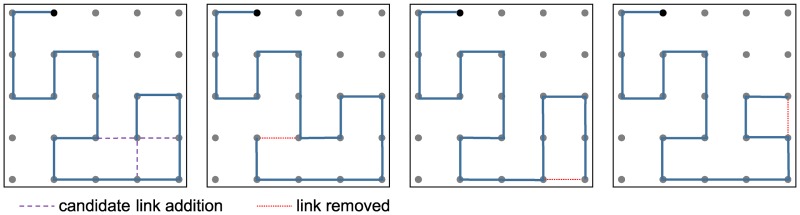
The backbite operator applied to an antenna starting at node 2 (black). Dashed lines in the leftmost design indicate the three links that may be added. The three diagrams to the right depict the different designs that result from each of these changes.

EAs are coarse grain optimizers, so require a local search mechanism in order to find locally optimal solutions. For the RFID problem, it is a non-trivial task to devise an effective local search scheme due to the black box that evaluates the characteristics of each design. Weis et al. [12] investigated a novel way of perturbing the ends of meander line antenna structures known as *backbite*, based on approaches for modifying SAWs [16]. The technique works by extending the end of the meander line to one of the (up to) three adjacent nodes, then deterministically removing an existing link so that no circuit is introduced. Fig 2 illustrates the process.

Each antenna design typically has three backbite-generated alternatives, as meander line endpoints are surrounded by up to three unoccupied segments. The process can be applied recursively to each alternative, and the *depth* of the recursion determines the total number of alternatives generated, which grows according to *O*(3^*d*^) where *d* is the depth. Weis et al. [12] considered depths between 5 and 25, as they were particularly interested in the impact of bends in antenna designs on quality (because their ACO algorithm incorporated a bias towards straight lengths). Lewis et al. [10] then incorporated the backbite operator as part of their multiobjective ACO algorithm, applying backbite with a fixed depth 3 to each of the 10 ACO-generated solutions at each iteration. These additional designs were then combined with the solutions produced by ACO before non-dominated sorting (see, e.g., Deb et al. [23]) was applied to filter out poor designs. In that previous work, the evaluation of backbite solutions did *not* count towards the 10,000 total allowed the ACO algorithm, since a comparison between algorithms was not a consideration in that work. As it is more typical to count all solution evaluations, that is the approach taken in the present work. Consequently, depths of 1 or 2 are considered, otherwise the number of solutions produced by the DE algorithm (and hence the number of iterations it is allowed) becomes extremely small.

The next section presents the DE algorithm for RFID antenna design and modifications introduced since its initial application to this problem, after which the modifications needed to incorporate backbite-based local search are explored.

## Differential evolution for RFID antenna design

Montgomery et al. [8] described the first application of DE to the multiobjective version of the RFID design problem. DE is a population-based search heuristic that operates in continuous domains and which has been applied successfully to many different problems [20]. Single-objective DE algorithms operate a generational model where, at each iteration, each solution is considered as a *target* for replacement by a newly generated solution. Adaptations of DE to MOO vary in their similarity to DE for single-objective optimization [24]. The heuristic solver used by Montgomery et al. [8] and subsequent work is a multiobjective DE/rand/1/exp algorithm that uses Pareto ranking to select between archived and newly generated solutions. This means that it retains the solution mutation mechanism of DE but uses general-purpose MOO mechanisms to manage the population and archive of known good solutions, in this case the non-dominated sorting component of Deb et al.’s [23] NSGA-II. Similar approaches include Madavan’s [25] Pareto-Based Differential Evolution (PBDE), which uses a DE/current-to-rand/1/bin algorithm and the non-dominated sorting and ranking of NSGA-II, Xue et al.’s [26] Multi-Objective Differential Algorithm (MODE), and Iorio and Li’s [27] Non-dominated Sorting Differential Evolution (NSDE), which is essentially NSGA-II with the mutation operator replaced by a DE variant and thus is most similar to the algorithm considered here and previously for the RFID design problem.

DE was chosen as the second mainstream heuristic to apply to this problem because, being designed for continuous domains, it represents a stark contrast to the previous ACO approach [8]. As the goal of minimizing resonant frequency is strongly related to antenna length, it was decided that solutions should represent circuit-free meander lines (as in the prior ACO), which precludes the use of solvers such as Binary DE (BDE) [28] and Binary Particle Swarm Optimization (BPSO) [29]. Related work using EO [7] confirmed this decision, since the expanded search space of a segment-based representation comes at the cost of search efficiency. Unless a binary solution representation is suitable, adapting continuous solvers to discrete problem domains is generally a non-trivial task (see Onwubolu and Davendra [30] for several examples). In a Cartesian grid of nodes there are two primary ways to describe a self-avoiding walk (SAW) from a given starting point. The first is in terms of the *absolute* direction of travel, often described using the cardinal directions (N)orth, (E)ast, (S)outh and (W)est. The second is in terms of the *relative* change in direction of travel given a common initial direction, which can be labeled (L)eft, (F)orward and (R)ight. A path of *k* steps with a fixed starting node may then be described by *k* symbols from either the NESW or LFR alphabets. Inserting an additional solution component to select the start node 1–*n* on one edge of the grid, a solution for a bounded grid of *n* × *n* nodes consists of *n*^2^ components: *n*^2^ − 1 directional instructions plus the starting node. Mapping these solution encodings to a continuous space can be done by dividing each dimension (of arbitrary size) by the number of alternative directions in the encoding. The DE algorithm presented here defines each dimension to be in the range [0, 3].

**Fig 3:**
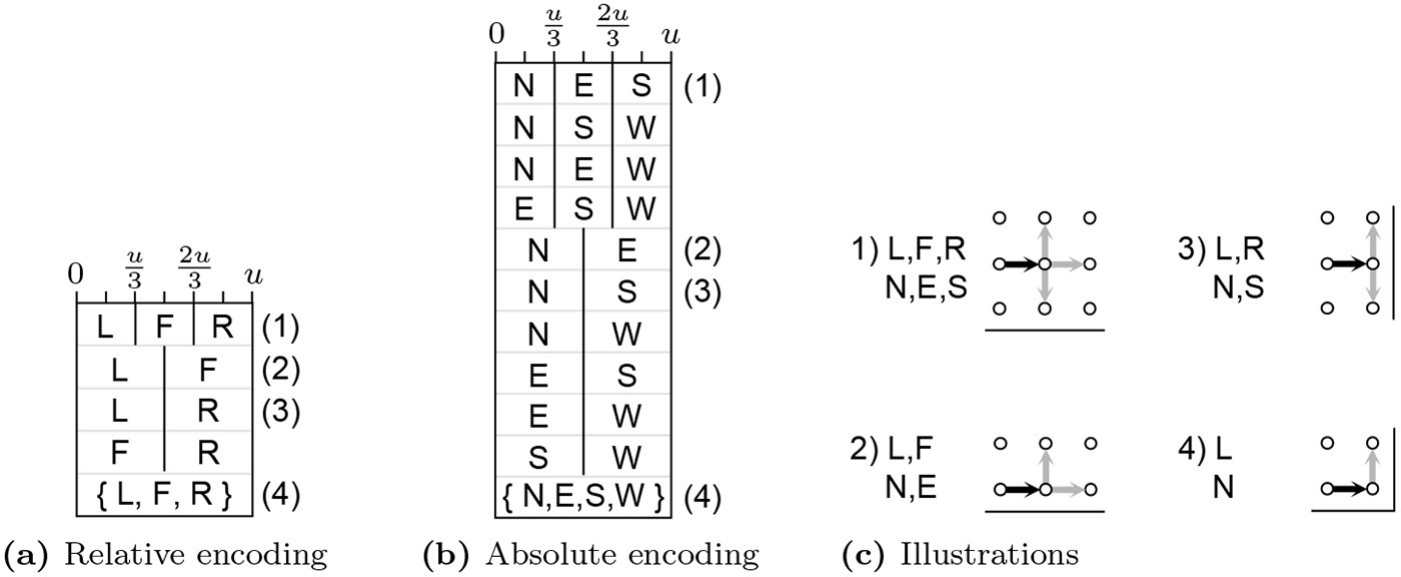
Enumeration of possible assignments of regions within a dimension to direction encoded given available directions of travel with the (a) relative and (b) absolute schemes, with construction scenarios illustrated in (c). The sets of directions {L, F, R} and {N, E, S, W} in (a) and (b) correspond to those cases when there is only one available direction in which to extend a path and hence that direction will be chosen regardless of the solution component’s current value.

Like their discrete counterparts, such encodings still permit sequences of instructions that produce short paths that cannot be extended without crossing themselves or leaving the design space. As longer paths are preferred, Montgomery et al. [8] adaptively decode each dimension such that the regions within that dimension are allocated only to the available directions of travel: options are assigned to regions in the same order as before, but allocated additional space. Fig 3 enumerates the regions within each dimension that may correspond to the instructions from both encodings, depending on the available options at a given time. This adaptive real-valued representation has since been expanded into a family of *adaptive generative representations*, suitable for other discrete problems that can be modeled as a series of constructive steps [22, 31].

Montgomery [21] evaluated the relative effectiveness of the four alternative representations that result from encoding either the *absolute* or *relative* direction of travel and whether or not solutions are decoded adaptively, finding that:

- adaptively interpreting solutions is necessary for both evolving and maintaining longer antenna designs; and
- the *relative* encoding, used in the first application of DE to this problem, produces superior performance to the *absolute* representation, both when evolving for antenna length alone and when solving the bi-objective antenna problem.

These results strongly suggest that the features of the representation (adaptively interpreting the relative direction of travel) have a stronger influence on search performance than the particular continuous solver used. Consequently, DE is used as a well-performing exemplar continuous solver.

Hence, the remainder of this paper uses the adaptive, relative encoding as the solution representation. The DE algorithmic variant used is DE/rand/1/exp algorithm, which was selected because the DE/rand/1/* family of algorithms is both widely-used and effective [32], while the *exp* crossover takes contiguous components from an intermediate candidate solution during crossover and is thus more likely to preserve antenna structure. Montgomery et al.’s [8] initial testing confirmed that it was more effective than DE/rand/1/bin. The algorithm uses crossover *Cr* = 0.99 and difference vector scale factor *F* = 0.8. While the initial work used a population size of 100, a later refinement introduced an external solution archive (described in the next section) which allows a smaller working population of 50 to be used [9].

### Extending the front using biased population selection

Although RFID antenna design is posed as a MOO problem, not all regions of the Pareto front are of equivalent interest, with solutions of lower resonant frequency (yet the highest efficiency possible) preferred. Most previous applications of heuristic solvers to this problem aimed to achieve good approximations of the (unknown but theoretically predicted) Pareto front without incorporating this preference. In order to encourage exploration of (near-)Pareto optimal solutions with lower resonant frequency, the earliest DE work [8] tested a minimum length constraint, which declared any antenna less than half the maximum length to be infeasible, and hence dominated by any longer solution. The constraint was placed on antenna length rather than on objective value because very short antennas have a tendency to be highly efficient. As that version of the algorithm used a single population of 100 individuals to both generate new solutions and represent the archive of best solutions, these short antennas, once generated, might never be replaced as they were non-dominated in the efficiency objective. The use of the minimum length constraint was successful in increasing the proportion of solutions with low resonant frequency for grid sizes up to 8 × 8, but retarded the search in larger grid sizes.

By modifying the algorithm to maintain a separate solution archive, able to grow beyond the size of the working population, other methods of directing the search become possible. Recently, the authors [9] investigated the utility of biasing selection of individuals in the working population from the archive such that low resonant frequency antennas were preferred. The specific approach, an extension of objective weighting functions proposed by Zitzler et al. [33] and Friedrich et al. [34], allows the search to be directed to different regions of the objective space. The bias is incorporated into the *crowding distance* calculation of the non-dominated sorting procedure and increases the apparent crowding distance (hence, making solutions more attractive).

In the present DE algorithm, when the archive is larger than the working population and no bias is being used, solutions are drawn from it with uniform probability. However, when the bias is in effect, solutions are drawn in non-increasing order of their crowding distance, which incorporates the bias weighting and thus biases the population used to produce the next generation of candidate solutions. The algorithm variant with a bias is denoted by DE_*bias*_ hereafter.

Although the bias function introduced by Montgomery et al. [9] can be varied to favour regions of the objective space between the two extremes, they found that a bias completely towards *f*_0_ is most effective. Overall, they found that the use of biased archive selection produces the best results in terms of number of solutions, quality of the front (and its likely proximity to the true Pareto front), and ability to focus on antenna designs with low resonant frequency. The impact of this algorithm component on solution quality on its own and in combination with local search is investigated in the penultimate section of this paper.

## Integrating discrete local search with a continuous solver

Incorporating the backbite local search operator into the DE-based search presents a number of challenges. Chiefly, the local search is conducted in the discrete space of antennas, but if improved solutions are to re-enter the DE population then they must be converted back into real-valued vectors. While in the algorithm presented thus far there exists a unique and deterministic mapping from real-valued vectors to antenna designs (see Fig 3), for each antenna design there exists an extremely large number of alternative vectors (finite in number due to limitations in the resolution of floating-point numbers). While this problem could be obviated by allowing backbite-generated solutions into the solution archive, but excluding them from further evolution by DE, this would prevent the DE search from being able to further refine them. If backbite-generated solutions are to be incorporated into the archive and working population then two key questions must be addressed:

1. Given that each backbite-generated solution has an ultimate parent that was defined by a DE solution vector, should the new solution vector be produced by *adapting* the values of that parent (such that they encode for the modified design) or by *regenerating* a solution vector from scratch? (Note that in both cases the parent solution is not modified.)
2. For each dimension that is modified (adjusted or regenerated), should this be done *deterministically* or *randomly*?

Underlying both questions is the issue of population diversity, since solutions produced by backbite may not replace the solutions they are based on. As a normal local search modifies some original solution, the adaptive approach seems plausible, but is guaranteed to leave many vector components unchanged, only modifying those needed to represent the modified antenna. Conversely, a completely new vector may represent a very similar antenna very differently from the original solution; diversity is maintained but the vector’s evolutionary history is lost. Modifying or generating vector components deterministically has the benefit that changes are predictable, but will also lead to a reduction in diversity as more vector components in the population take on one of a limited set of alternative values.

Given the choices in points 1 and 2 above, the four alternative schemes have been realized as follows. For each vector component, the available directions of travel in the antenna it represents are enumerated and used to establish the bounds for the region corresponding to the selected direction, denoted (*l*_*dir*_, *u*_*dir*_). In the adaptive scheme, if the parent component encodes for the new direction then it is copied unchanged (even if, due to epistatic effects, the direction in the new solution happens to be different from the parent). In the regenerating scheme, a new value is always generated. New values are determined in the following manner:

- When *adapting* the original solution vector *deterministically* the new value is placed 10% of the way within the range (*l*_*dir*_, *u*_*dir*_), nearest its original value, which represents a small change that is also somewhat robust under future modification. Given the range [0, 3], possible component values are consequently restricted to {0.9, 1.1, 1.9, 2.1} when there are three options and {1.85, 2.15} when there are two (the value will never be changed when there is only one available direction).
- When *regenerating* the solution vector *deterministically* the new value is placed at the midpoint of the range, limiting values to {0.5, 1.5, 2.5} when there are three options and {0.75, 2.25} when there are two.
- When either adapting or regenerating the solution vector *randomly*, each new value is selected with uniform probability to lie within the middle 99% of the valid range (to avoid boundary conditions).

In all experiments reported in the remainder of this work each trial is allowed 10,000 solution evaluations (200 iterations). Experiments were conducted on a multi-core system allowing eight solutions to be evaluated in parallel. This leads to runtimes of approximately 1 hour for the 5 × 5 problem, increasing linearly to 12 hours for the 10 × 10 problem. Antenna evaluation using NEC is the dominant operation, taking up to 90 seconds for a full-length antenna on the 10 × 10 grid due to the larger number of antenna segments. The total runtime of the DE is generally under three minutes, even for a 12-hour run. Maximum speedup could be achieved by evaluating all child solutions in parallel, if the host hardware allows this. As in the prior ACO algorithm, the backbite operator is applied to all 50 DE solutions produced at each iteration, resulting in up to 200 designs with depth 1 or 650 designs with depth 2 (although in practice neither search can generate the maximum number of alternative designs).

The complete algorithmic framework of the DE system for RFID antenna design is presented in Algorithm 1, and is supported by the NEC evaluation and non-dominated sorting operations, presented in Algorithms 2 and 3. No algorithm is presented for backbite, whose behaviour is defined in the previous section. Note that pseudocode in these algorithms describes the logical behaviour of the system, not the precise implementation in software.

**Algorithm 1** Multi-objective Differential Evolution for RFID Antenna Design

**procedure** DEforRFID(*n*, *N*, *bias*, *ls*, *bbdepth*)  ▷ grid size *n*, population size *N*

  ▷ objective *bias*, local search reintegration strategy *ls*, backbite depth *bbdepth*

 **let** *archive* ← *N* uniformly randomly initialized vectors of length *n*

 For each solution *vector* in *archive* call EvaluateAntenna(*vector*)

 *archive* ← NondominatedSorting(*archive*, *N*, *bias*)

 **while** function evaluations below limit **do**

  **if** |*archive*| > *N* **then**  ▷ Select working population for this iteration

   **if** using *bias* **then**

    **let** *pop* ← Select first *N* solutions from previously sorted *archive*

   **else**

    **let** *pop* ← *N* distinct, randomly selected solutions from *archive*

   **end if**

  **else**

   **let** *pop* ← *archive*

  **end if**

  **let** *children* ← Apply **DE/rand/1/exp** to *pop*

  **if** applying local search **then**  ▷ Generate additional children using backbite

   **let** *bbnodepaths* ← Backbite(*children*, *bbdepth*)

   **let** *bbchildren* ← Convert *bbnodepaths* to vectors using *ls* strategy

   *children* ← *children* ∪ *bbchildren*

  **end if**

  For each solution *vector* in *children* call EvaluateAntenna(*vector*)

  *archive* ← *archive* ∪ *children*

  *archive* ← NondominatedSorting(*archive*, *N*, *bias*)

 **end while**

 **return** *archive*

**end procedure**

**Algorithm 2** NEC-based Antenna Evaluation Procedure

**function** EvaluateAntenna(*vector*)

 **let** *nodepath* ← Decode *vector* instructions

 **if** *nodepath* in cache **then**

  **let** *f*_0_, *η* ← Retrieve values from cache

 **else**

  Construct NEC input file for *nodepath*

  **let** *f*_0_, *η* ← Execute external **NEC simulator**

 **end if**

 **return** *f*_0_, *η*

**end function**

**Algorithm 3** Non-dominated sorting incorporating objective bias

**function** NondominatedSorting(*archive*, *N*, *bias*)

 **let** *archive*′ ← Select and remove all non-dominated solutions from *archive*

 **if** *archive* is empty then  ▷ All solutions are front 1; allow size to exceed *N*

  Calculate crowding distances (including *bias*) for solutions in *archive*′

  Sort *archive*′ by non-increasing order of crowding distance

 **else**

  **while** |*archive*′| < *N* **do**

   **let** *front* ← Select and remove all non-dominated solutions from *archive*

   **if** |*archive*′| + |*front*| > *N* **then**  ▷ Select least crowded solutions

    Calculate crowding distances (including *bias*) for solutions in *front*

    Sort *front* by non-increasing order of crowding distance

    Truncate *front* to first *N* − |*archive*′| solutions

   **end if**

   *archive*′ ← *archive*′ ∪ *front*

  **end while**

 **end if**

 **return** *archive*′

**end function**

The performance of the DE algorithm with the four local search variants was examined on the comparatively small 7 × 7 problem (selected because it is large enough for differences to be apparent, while each run, parallelized to evaluate eight solutions simultaneously, takes less than four hours to complete using the NEC evaluation package [14]). Eight algorithm combinations were considered: each of the four schemes for mapping antennas back to real-vectors described above with a backbite depth of either 1 or 2. Twenty randomized trials were conducted for each combination.

Performance was measured using the hypervolume (HV) metric [35], with objective values (*f*_0_, *η*) normalized within the region specified by the (unachievable) utopia point (350, 100) and nadir point (2250, 48). Although this region excludes up to 1% of discovered solutions with extremely high resonant frequency it includes all others observed in this study. Reported HV values have been multiplied by 100 for readability, so represent the percentage of the bounded region covered by a solution set.

**Fig 4:**
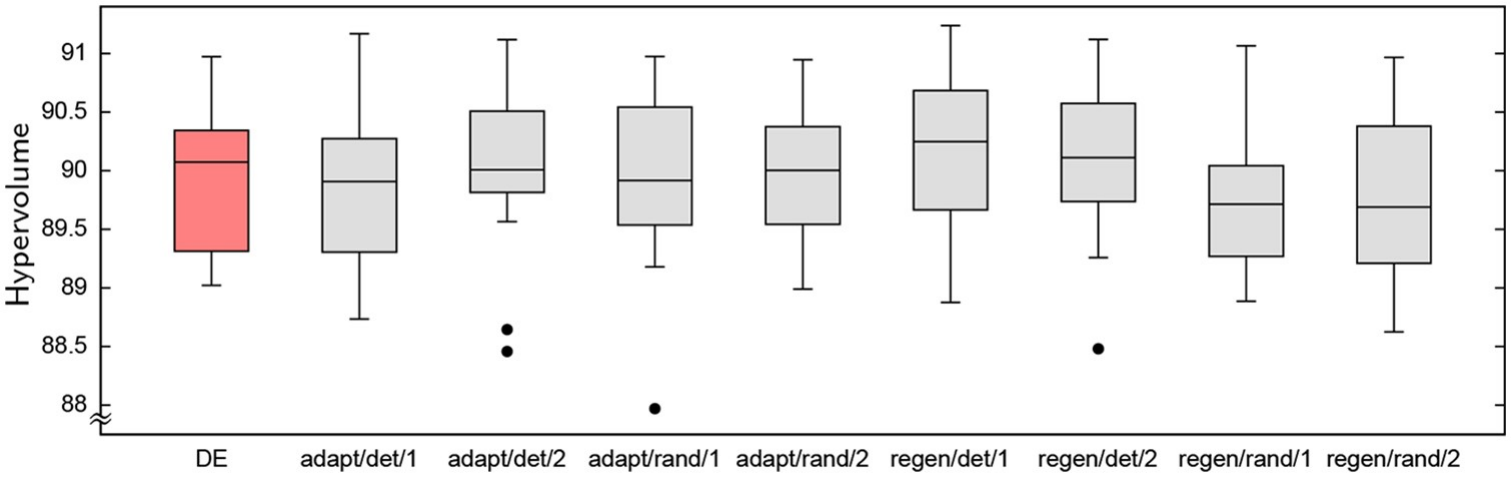
Distributions of HV for standard DE and DE with alternative local search approaches, on the 7 × 7 problem. Variants are labeled by whether they *a*dapt the original solution vector or *r*egenerate a new vector, *r*andomly or *d*eterministically, and by the depth of the backbite search (1 or 2).

Fig 4 shows the distributions of hypervolume for each algorithm variant as well as the unmodified DE algorithm (for which 10 randomized trials were performed). While there is an apparent trend towards better performance in the adaptive approaches using a greater backbite depth, Mann-Whitney tests show most of the observed differences are not statistically significant. However, the poorer performance of randomly regenerating solutions *is* statistically significant compared to deterministically regenerating them (at the 5% level for backbite depth 1 and the 10% level for depth 2), and approaching statistical significance for others except for deterministically adapting solutions. None of the observed results is statistically significantly different from DE on the same problem size.

The final solution sets of the local search variants were further compared using the *C*-metric (see, e.g., Zitzler and Thiele [35]). Denoted *C*(*A*, *B*), the *C*-metric describes the proportion of points in the surface produced by *B* that are dominated by at least one point produced by *A*. Table 1 presents the average *C*-metric value produced by comparing each of the 20 trials with one local search against each of the 20 trials from another (i.e., the average over 400 *C*-metric measurements). This confirms the apparent differences in hypervolume shown in Fig 4 and suggests that deterministically regenerating solutions produces better outcomes than the other approaches. Conversely, *randomly* regenerating solutions appears to perform worst.

**Table 1:** Average *C*(*row*, *col*) for local search variants on 7 × 7 problem. Cell (*row*, *col*) corresponds to the average of *C*(*A*, *B*), the proportion of solutions produced by *B* that are dominated by solutions produced by *A*. Variants are labeled as in Fig 4. The last column is the row average.

	adapt				regen				*average*
	det/1	det/2	rand/1	rand/2	det/1	det/2	rand/1	rand/2	
adapt/det/1		0.323	0.307	0.338	0.221	0.253	0.306	0.307	0.294
adapt/det/2	0.296		0.297	0.323	0.212	0.240	0.298	0.294	0.280
adapt/rand/1	0.312	0.328		0.339	0.230	0.257	0.307	0.309	0.297
adapt/rand/2	0.287	0.301	0.289		0.205	0.233	0.284	0.281	0.269
regen/det/1	0.367	0.382	0.360	0.395		0.306	0.358	0.361	0.362
regen/det/2	0.368	0.380	0.358	0.392	0.273		0.359	0.364	0.356
regen/rand/1	0.278	0.289	0.280	0.306	0.197	0.225		0.268	0.263
regen/rand/2	0.280	0.290	0.283	0.307	0.197	0.224	0.277		0.265

In terms of the relative number of solutions produced by backbite compared to the normal DE mutation operator, the backbite search depth has the largest impact. When the depth is 1, typically 69% of solutions come from backbite and 31% from DE (meaning the number of DE *generations* is similarly truncated). When the depth is increased to 2, on average 80% of solutions evaluated come from backbite. For a given backbite depth, differences between variants in the number of solutions produced by backbite are mostly statistically significant (at the 1% level), although as the effect size is very small no conclusion can be drawn about the cause.

Each variant’s impact on solution diversity, measured as the proportion of dissimilar values within each solution dimension across the population, is as would be expected. Regenerating solution vectors randomly leads to the greatest diversity, followed by adapting solutions randomly and adapting solutions deterministically (where new vectors share some component values with their parent and new values are selected from a small set of alternatives). Regenerating solution vectors deterministically produces the lowest amount of variation across the population. The final solution sets produced when regenerating vectors deterministically are also somewhat more crowded, with solutions less evenly spread across the front (this result is statistically significant at the 1% level). Notably, however, algorithm performance appears to be inversely related to population diversity.

Given that these results are suggestive rather than conclusive, experiments in the next section combining biased archive selection and local search consider three local search variants with a backbite depth of 1: regenerate-deterministically (the apparent “best”), regenerate-randomly (the likely “worst”), and adapt-randomly (the “second best” in terms of *C*-metric performance and good hypervolume performance). Although a greater backbite depth produced better hypervolume performance in the adaptive variants, the average *C*-metric results for these are poorer and using the same backbite depth eliminates a confounding variable.

## Combining biased population selection with local search

On its own, the backbite operator can generate additional alternative designs similar to those produced by the DE algorithm, but there is no particular direction to its search apart from the selection pressure imposed by non-dominated sorting of solutions. Indeed, results presented in the previous section indicate that DE with the backbite operator performs similarly to standard DE, despite some variation between local search approaches. This section examines the efficacy of combining DE_*bias*_ with local search. The experimental setup is as follows: for each of the grid sizes 5 × 5 through 10 × 10, 10 randomized trials were performed with DE_*bias*_ incorporating one of the three selected local search variants, regenerate deterministically (denoted ${\text{DE}}_{bias}^{\text{r}/\text{d}/1}$), adapt randomly (denoted ${\text{DE}}_{bias}^{\text{a}/\text{r}/1}$) and the anticipated poorest combination of regenerate randomly (denoted ${\text{DE}}_{bias}^{\text{r}/\text{r}/1}$).

**Fig 5:**
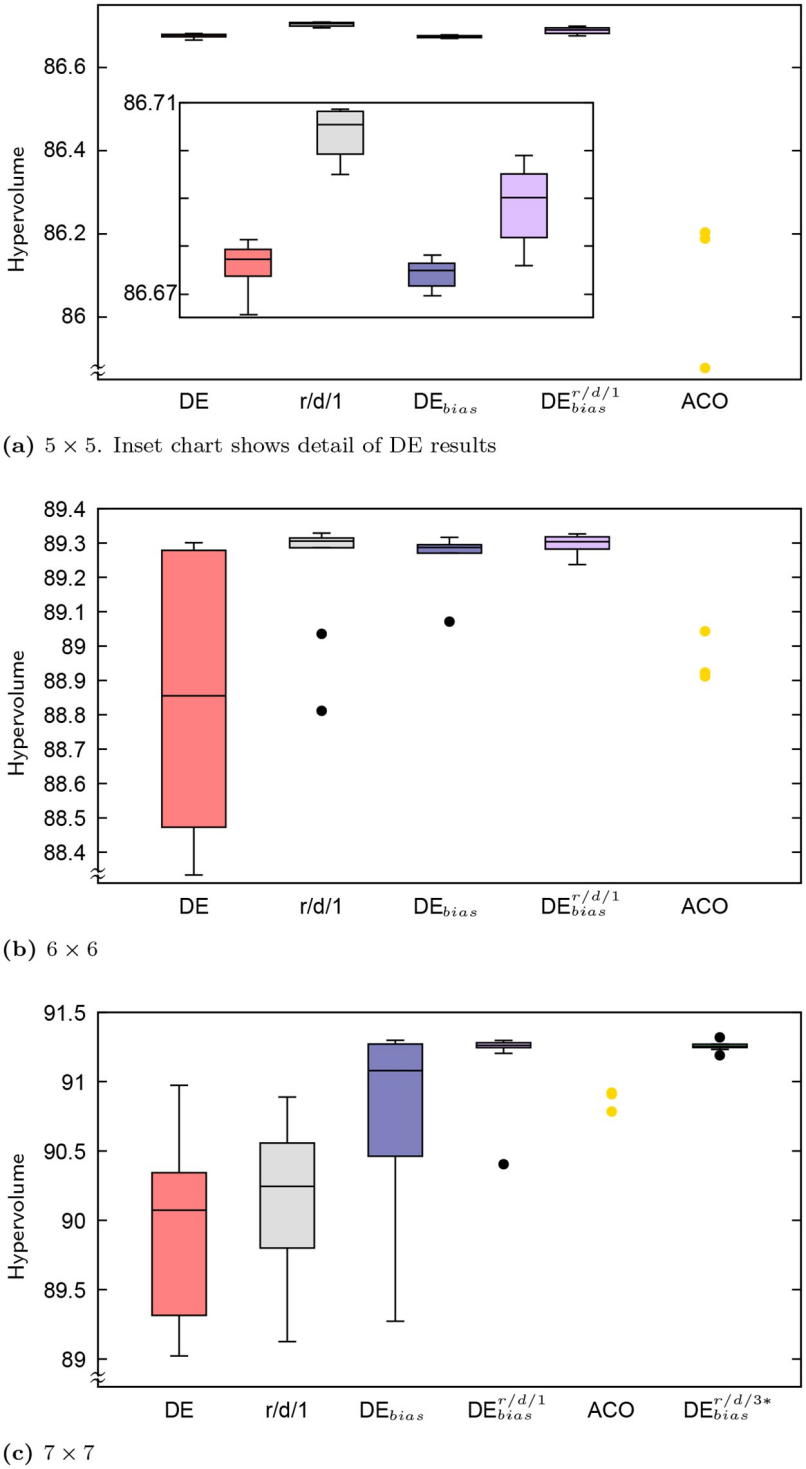
Distributions of HV across problem sizes 5 × 5 through 7 × 7 for DE, DE with the r/d/1 local search variant, DE_*bias*_, ${\text{DE}}_{bias}^{\text{r}/\text{d}/1}$ and ${\text{DE}}_{bias}^{\text{r}/\text{d}/3*}$. Also plotted are the HVs of the earlier ACO (using three different greediness settings) with local search.

**Fig 6:**
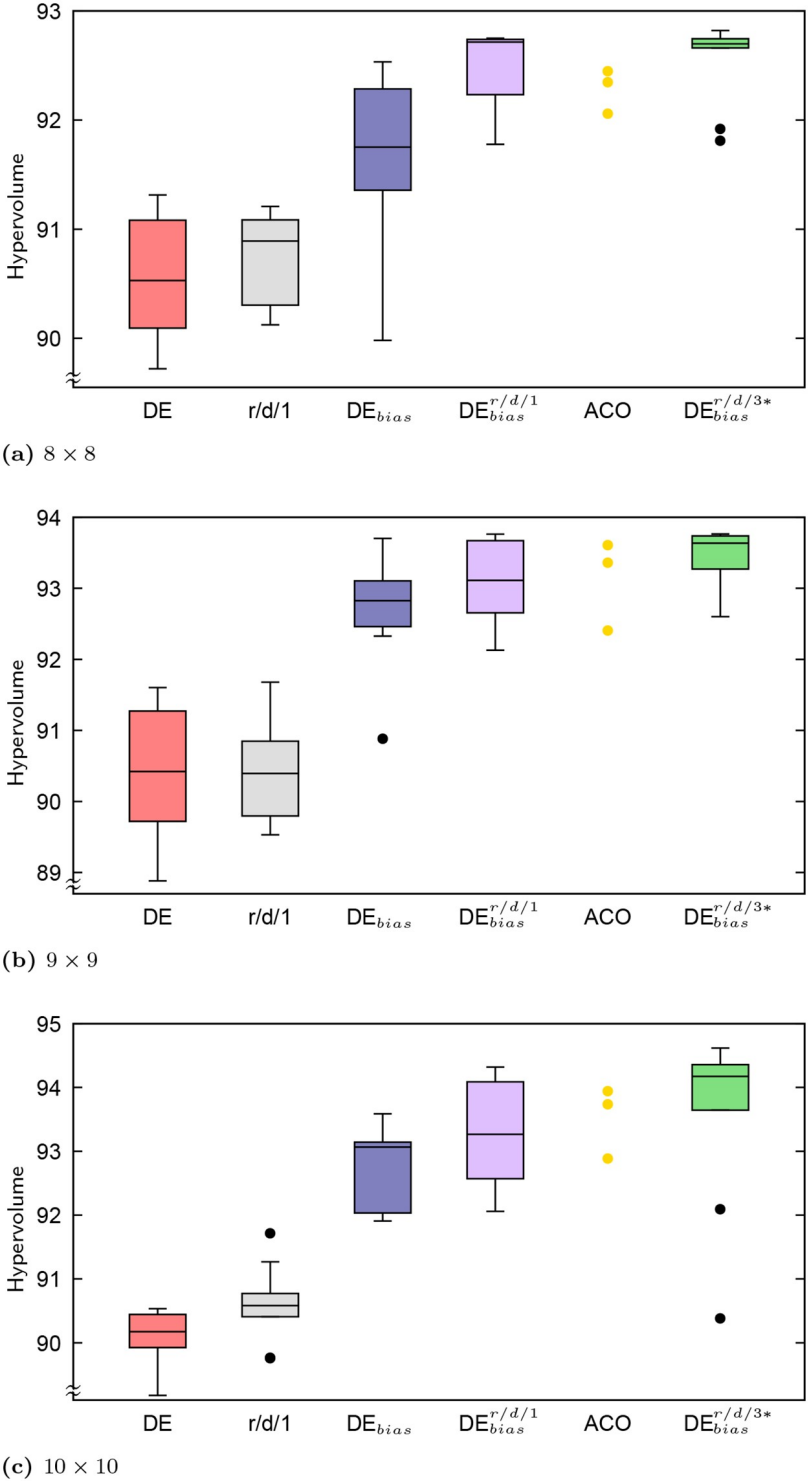
Distributions of HV across problem sizes 8 × 8 through 10 × 10.

Table 2 presents summary statistics for HV and the minimum *f*_0_ in the set of solutions produced, while Table 3 presents summary statistics for the total number of solutions in the final set of non-dominated solutions and the number of those solutions that have a “low” value of *f*_0_ ≤ 600 MHz. The best observed value within each metric and statistic (best, median or worst) is shown in bold. Also included in the table are results for the standard DE algorithm (control) and DE_*bias*_ without local search, which include data from five additional runs not included in the prior work by Montgomery et al. [9]. Distributions over HV for all grid sizes are also presented as box plots in Figs 5 and 6, which includes additional results for algorithms discussed below.

**Table 2:** Best, median and worst results for hypervolume and minimum *f*_0_ for DE_*bias*_ with different local search approaches, a standard DE control, and DE_*bias*_.

		Hypervolume			Minium *f*_0_		
		best	median	worst	best	median	worst
5 × 5	r/d/1	**86.7**	**86.7**	**86.7**	**574**	**574**	**574**
	a/r/1	**86.7**	**86.7**	**86.7**	**574**	**574**	**574**
	r/r/1	**86.7**	**86.7**	**86.7**	**574**	**574**	**574**
	control	**86.7**	**86.7**	**86.7**	**574**	**574**	**574**
	biased	**86.7**	**86.7**	**86.7**	**574**	**574**	**574**
6 × 6	r/d/1	**89.3**	**89.3**	**89.2**	**514**	**514**	**514**
	a/r/1	**89.3**	**89.3**	**89.3**	**514**	**514**	**514**
	r/r/1	**89.3**	**89.3**	**89.3**	**514**	**514**	**514**
	control	**89.3**	88.9	88.3	**514**	525	536
	biased	**89.3**	**89.3**	89.1	**514**	**514**	520
7 × 7	r/d/1	**91.3**	**91.3**	**90.4**	**464**	**464**	**486**
	a/r/1	**91.3**	91.0	89.3	**464**	469	510
	r/r/1	**91.3**	**91.3**	**90.4**	**464**	**464**	**486**
	control	91.0	90.1	89.0	471	493	518
	biased	**91.3**	91.1	89.3	**464**	469	508
8 × 8	r/d/1	**92.7**	**92.7**	**91.8**	**420**	**420**	**448**
	a/r/1	**92.7**	92.1	89.8	**420**	436	496
	r/r/1	92.5	91.9	90.2	426	444	490
	control	91.3	90.5	89.7	457	478	497
	biased	92.5	91.8	90.0	427	443	492
9 × 9	r/d/1	**93.8**	**93.1**	**92.1**	**383**	**402**	**423**
	a/r/1	93.5	92.3	90.1	392	424	470
	r/r/1	93.3	92.5	89.7	395	419	490
	control	91.6	90.4	88.9	449	476	512
	biased	93.7	92.8	90.9	387	416	463
10 × 10	r/d/1	**94.3**	**93.3**	**92.1**	**352**	**385**	**416**
	a/r/1	93.5	91.1	90.2	388	446	475
	r/r/1	92.8	91.9	89.9	405	430	482
	control	90.5	90.2	89.2	462	476	496
	biased	93.6	93.1	91.9	390	404	431

**Table 3:** Best, median and worst total solution set size and number of low *f*_0_ solutions for DE_*bias*_ with different local search approaches, a standard DE control, and DE_*bias*_.

		total solutions…			…with *f*_0_ ≤ 600		
		best	median	worst	best	median	worst
5 × 5	r/d/1	137	121	117	**12**	11	**10**
	a/r/1	139	131	85	**12**	11	6
	r/r/1	**147**	**136.5**	**125**	**12**	**11.5**	**10**
	control	103	101	98	9	9	7
	biased	99	96.5	93	9	9	8
6 × 6	r/d/1	**210**	180	133	**81**	**73**	**42**
	a/r/1	211	178.5	137	75	61	39
	r/r/1	203	**182.5**	**158**	63	57.5	33
	control	153	140.5	115	42	22.5	12
	biased	200	162.5	133	65	57.5	30
7 × 7	r/d/1	292	**243.5**	**200**	**169**	**131**	**110**
	a/r/1	273	232.5	193	129	105	61
	r/r/1	**312**	221	190	154	81	49
	control	201	145	122	47	25.5	17
	biased	245	197	141	146	85.5	37
8 × 8	r/d/1	**349**	**312.5**	**291**	**214**	**186.5**	**156**
	a/r/1	297	274.5	224	158	138.5	59
	r/r/1	335	221	186	149	70	40
	control	217	176.5	136	42	26	16
	biased	301	237.5	189	173	121.5	79
9 × 9	r/d/1	**408**	**378.5**	**320**	**249**	**228**	**159**
	a/r/1	364	295.5	250	185	157	84
	r/r/1	320	262.5	186	120	90.5	43
	control	217	179.5	129	50	33	15
	biased	291	269	192	176	131	61
10 × 10	r/d/1	**455**	**390.5**	**349**	**294**	**225**	**145**
	a/r/1	379	310	259	201	138	89
	r/r/1	308	264.5	211	106	87	52
	control	221	188.5	170	27	24.5	17
	biased	312	254.5	204	162	108	80

Across most metrics ${\text{DE}}_{bias}^{\text{r}/\text{d}/1}$ does indeed outperform the other DE_*bias*_ with local search variants and the control and DE_*bias*_ algorithms, with differences becoming more apparent as problem size grows. Due to the small amount of data (10 samples for each metric), two-tailed Mann-Whitney tests were performed on all pairs of algorithm combinations, revealing many of the differences to be statistically significant at the 1% or 5% levels. In particular:

- ${\text{DE}}_{bias}^{\text{r}/\text{d}/1}$ achieves a better HV than the other local search variants for problem sizes 9 × 9 and 10 × 10, and is better than ${\text{DE}}_{bias}^{\text{r}/\text{r}/1}$ on 8 × 8. It is also better than DE on all problem sizes and better than DE_*bias*_ on grids 8 × 8 and above.
- The minimum *f*_0_ achieved by ${\text{DE}}_{bias}^{\text{r}/\text{d}/1}$ is typically better than the other local search variants, always better than DE, and better than DE_*bias*_ on grids 7 × 7 and above, although for grids 9 × 9 and 10 × 10 this is only statistically significant at the 10% level.
- For grid sizes 7 × 7 and above, the observed differences in final solution set size are statistically significant at the 1% level in most cases (with some exceptions on the 7 × 7 problem). This trend is more pronounced when considering the number of solutions with “low” *f*_0_.
- Differences between the selected “second best” local search variant ${\text{DE}}_{bias}^{\text{a}/\text{r}/1}$ and the likely poorest variant ${\text{DE}}_{bias}^{\text{r}/\text{r}/1}$ were only statistically significant when considering the size of the final solution set and number of solutions with “low” *f*_0_ (for grid 8 × 8 and above).

Unlike in the initial comparison of local search variants on the 7 × 7 problem, when combined with DE_*bias*_ the differences in the number of solutions produced by backbite versus DE are both moderate in size and statistically significant across problem sizes at the 1% level (except in two cases which were significant at the 5% level). On the 10 × 10 problem, the median number of DE-produced solutions for ${\text{DE}}_{bias}^{\text{r}/\text{d}/1}$, ${\text{DE}}_{bias}^{\text{a}/\text{r}/1}$ and ${\text{DE}}_{bias}^{\text{r}/\text{r}/1}$ represented 27%, 28% and 31% of the total, respectively. Although the magnitude of difference is small, this effect is noteworthy because the ratio is determined solely by the number of alternative solutions that backbite can produce, which is influenced by the position of each antenna’s end on the grid. This suggests there is some effect of the interaction between the local search—really, solution reintegration—approach and DE search that leads to quite distinct antenna designs.

### Comparing major variants and ACO

Figs 5 and 6 present the distributions of HV values across problem sizes as boxplots, for most of the DE variations described previously [8, 9] and in this work: DE, DE with the deterministically regenerated solutions and backbite depth of 1 (labeled r/d/1), DE_*bias*_, and ${\text{DE}}_{bias}^{\text{r}/\text{d}/1}$. Two other HV result sets are plotted for comparison: those of Lewis et al.’s [10] ACO with local search, which applied an Ant Colony System algorithm (see Dorigo and Stützle [17] for details) using three different greediness settings (*q*_0_ ∈ {0.1, 0.5, 0.9}), and from re-running DE_*bias*_ with r/d/1 local search under more similar experimental conditions to the ACO. The ACO algorithm used a population of 10 ants and was run for 1,000 iterations (hence, 10,000 function evaluations total for the ACO). In addition to these 10,000 solutions, the ACO algorithm applied the backbite operator with depth 3 to each solution produced at each iteration, leading to up to 27 additional solutions per ACO solution that were evaluated but not counted in the 10,000 limit. In contrast, the standard DE algorithm presented here counts backbite solutions within its budgeted function evaluations, which is thus an order of magnitude less than that allowed the ACO algorithm. While the observed runtimes for the ACO are not known (and would not be comparable given differences in hardware used), the two algorithms have equivalent algorithmic complexity given how new solutions are constructed, backbite is used to generate additional solutions, and non-dominated sorting is used for filtering at each iteration. Biased archive selection, employed by DE_*bias*_ and its derivatives, but not by the ACO, is incorporated into the crowding distance calculation of non-dominated sorting. For both algorithms runtime is dominated by evaluating solutions with NEC. To provide a suitable comparison, DE_*bias*_ with local search and regenerate/deterministic solution reintegration was re-run under the same conditions as the ACO with backbite depth 3 and backbite-generated solutions excluded from the 10,000 solution evaluation limit. This variant, denoted ${\text{DE}}_{bias}^{\text{r}/\text{d}/3*}$, was applied to problem sizes 7 × 7 and up.

Results for the knapsack-based EO approach of Gomez-Meneses et al. [7] are not included because, exploring the much larger space of segment-based antennas, they are significantly poorer than all DE variants and the ACO. For instance, on the 7 × 7 problem, that algorithm produced only six antennas with *f*_0_ below 1000 MHz, with the lower extreme of its attainment surface an attenna with *f*_0_ = 662 MHz, *η* = 93%.

The solution sets for the prior ACO approach were re-evaluated using the same implementation of the NEC simulation software used by the DE algorithm. This evaluates *f*_0_ at the finer resolution of 1 MHz instead of 10 MHz and also treats the *side lobes* of an antenna’s radiation pattern somewhat differently. This resulted in some solutions becoming dominated and hence excluded from the HV calculations and the attainment surfaces presented later. The implementation of NEC used in this work was, however, obtained from one of the authors of Lewis et al.’s [10] work, and the source code is available with the accompanying result dataset.

**Fig 7:**
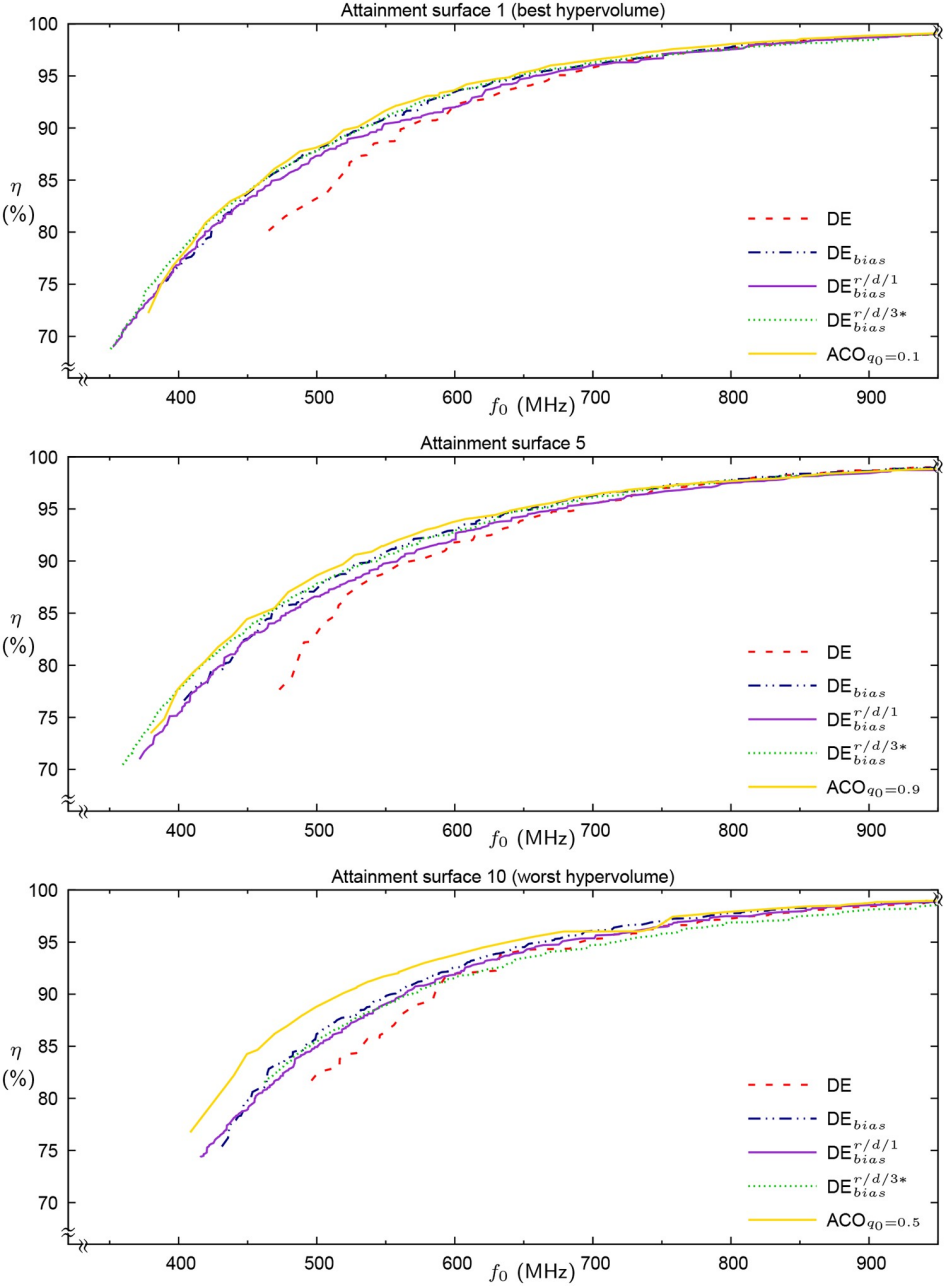
First (best), fifth (near median) and last summary attainment surfaces for the 10 × 10 problem using normal DE, DE_*bias*_, ${\text{DE}}_{bias}^{\text{r}/\text{d}/1}$ and ${\text{DE}}_{bias}^{\text{r}/\text{d}/3*}$. Best, second best and worst of the three ACO result sets are plotted independently, in that order.

To visualize the relative performance of these approaches, Fig 7 shows the best, fifth-best (i.e., near median) and worst attainment surfaces, ranked by hypervolume, for the DE control, DE_*bias*_, ${\text{DE}}_{bias}^{\text{r}/\text{d}/1}$ and ${\text{DE}}_{bias}^{\text{r}/\text{d}/3*}$ on the 10 × 10 problem. As only three result sets are available for the prior ACO approach, these fronts are plotted across the three charts, in decreasing order of hypervolume. In the case of the 10 × 10 problem, the least greedy approach was best, followed very closely by the greediest approach, while the intermediate greediness setting was poorest.

Considering the DE variants, merely adding backbite local search is insufficient to obtain consistently improved results. Montgomery et al. [9] demonstrated previously that adding a selection bias towards *f*_0_ strongly improves the algorithm’s performance, while the present results indicate that combining the bias with a suitable mechanism for reintegrating backbite-generated solutions into the population can improve it further.

Compared with the prior ACO results, ${\text{DE}}_{bias}^{\text{r}/\text{d}/1}$ demonstrates superior performance for grid sizes up to 8 × 8. On the 9 × 9 problem, two data points for ACO sits within the third quartile of observed DE results, while the other is in the bottom quartile, which suggests that the DE is at least as good. Similarly on the most challenging 10 × 10 problem, where the hypervolume measures of two of the ACO result sets are towards the upper end of the third quartile of DE results, with its third result set lying towards the bottom of the second quartile. Although there is insufficient data to perform tests for statistical significance, these comparative results are suggestive of equivalent performance by the DE algorithm, given considerably fewer function evaluations.

The expanded DE with local search runs of ${\text{DE}}_{bias}^{\text{r}/\text{d}/3*}$ on problem sizes 7 × 7 and up are suggestive of a positive impact on hypervolume, minimum *f*_0_ achieved and on number of solutions produced. Median outcomes for these measures are presented in Table 4 for both ${\text{DE}}_{bias}^{\text{r}/\text{d}/3*}$ and ACO. The comparatively low solution count for ACO is a product of both small original result set sizes (medians 86, 87, 190, 130 for grid sizes 7–10, respectively) and of the re-evaluation of solutions causing some solutions to become dominated. Applying Mann-Whitney tests and comparing ${\text{DE}}_{bias}^{\text{r}/\text{d}/1}$ and ${\text{DE}}_{bias}^{\text{r}/\text{d}/3*}$, the differences between hypervolume are not statistically significant, although approaching significance on the 9 × 9 and 10 × 10 problems. Differences in minimum *f*_0_ achieved are only statistically significant on the 9 × 9 problem (*p* = .02). The additional depth (and amount) of backbite search does lead to a larger number of solutions, a result that is statistically significant at the 1% level (*p* < .001). Comparing ${\text{DE}}_{bias}^{\text{r}/\text{d}/3*}$ against the middle-performing ACO, the results are suggestive of better performance on all three measures. However, the extended runs are not always effective, with at least two trials on 8 × 8 and 10 × 10 performing quite poorly, with the additional exploration afforded by the deeper backbite search unable to allow the algorithm to make better progress toward to the true Pareto front.

**Table 4:** Median result for hypervolume (HV), minimum *f*_0_, total solution set size and number of low *f*_0_ solutions for ${\text{DE}}_{bias}^{\text{r}/\text{d}/3*}$ and the prior ACO.

Size	Algorithm	HV	min *f*_0_	Solution count	
				total	with *f*_0_ ≤ 600
7 × 7	${\text{DE}}_{bias}^{\text{r}/\text{d}/3*}$	91.3	464	262	141.5
	ACO	90.9	469	70	18
8 × 8	${\text{DE}}_{bias}^{\text{r}/\text{d}/3*}$	92.7	420	382	260.5
	ACO	92.3	428	69	21
9 × 9	${\text{DE}}_{bias}^{\text{r}/\text{d}/3*}$	93.6	383	551	396
	ACO	93.4	397	110	28
10 × 10	${\text{DE}}_{bias}^{\text{r}/\text{d}/3*}$	94.2	359	706.5	529.5
	ACO	93.7	378	87	28

**Fig 8:**
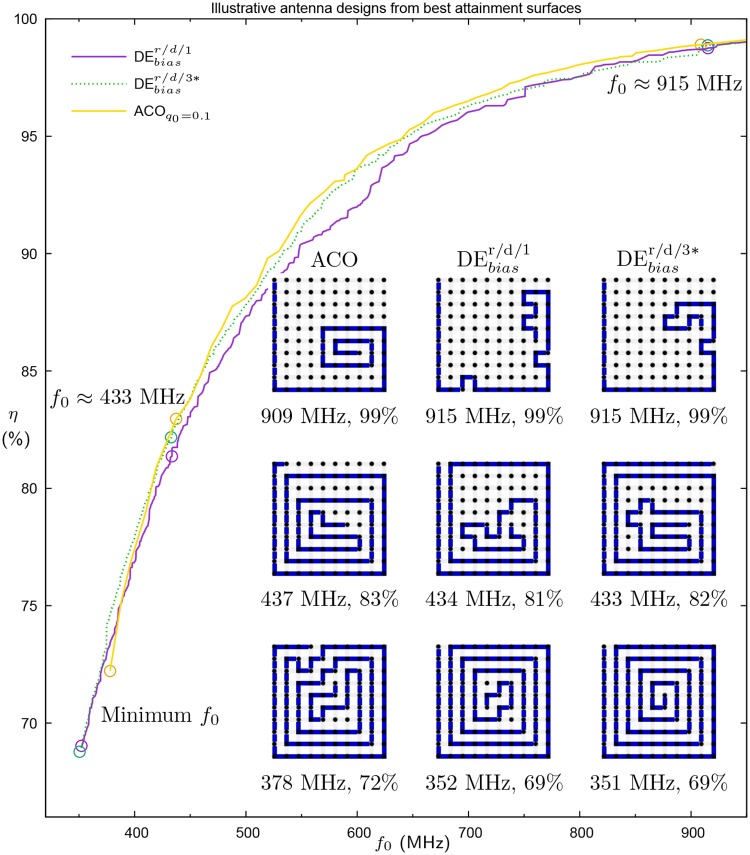
Illustrative antenna designs produced by the best run (based on HV) of ACO, ${\text{DE}}_{bias}^{\text{r}/\text{d}/1}$ and ${\text{DE}}_{bias}^{\text{r}/\text{d}/3*}$. The bottom row comprises antennas with lowest *f*_0_, the middle row those with *f*_0_ nearest 433 MHz and the top row those with *f*_0_ nearest 915 MHz. The location of each antenna is circled on the attainment surfaces (reproduced from Fig 7).

Fig 8 depicts illustrative antenna designs generated by ACO, ${\text{DE}}_{bias}^{\text{r}/\text{d}/1}$ and ${\text{DE}}_{bias}^{\text{r}/\text{d}/3*}$. The antennas were selected from the run of each algorithm with the best hypervolume and represent the antennas with the minimum *f*_0_ achieved or with *f*_0_ closest to one of two standard RFID frequencies 433 MHz and 915 MHz [36, 37]. While some antenna designs end at points that could be extended further, this is the result of applying the backbite operator to designs that had reached dead ends. Backbite was not intended to allow the algorithm to “recover” from such designs, but to explore additional designs that are similar to those produced by the DE (or ACO). A common feature of the designs is a spiral pattern, a perfect example of which was generated by the DE with expanded application of backbite. A tendency to produce spirals (as opposed to other, human-engineered designs such as the “plough”), appears to have become canalised (effectively fixed) within the population. It also fits with prior work by Montgomery and Ashlock [31], which examined the shape of evolved SAW designs, finding that constructing a spiral is correlated with longer paths as it leaves a greater amount of the design space accessible during antenna construction.

Overall, the present findings demonstrate clearly that, by combining a bias towards lower resonant frequency and a moderate level of backbite search (conducted *within* the function evaluation limit), DE can be seen as the preferred approach. Further, under the more relaxed conditions of deeper backbite search, conducted outside the function evaluation limit, the approach can outperform a prior ACO algorithm for this problem. Broader implications are discussed in the next section.

## Conclusions and recommendations

The present work is part of a broad effort to apply heuristic search techniques to discover the realistic limitations on the performance of planar RFID antennas. While theoretical limits are known [5], it is only by simulating (or constructing) alternative designs that the actual boundaries will be found. Efforts in this area span a variety of heuristic approaches, including discrete techniques such as ACO through to continuous solvers like differential evolution and particle swarm optimisation (PSO).

Solvers that work canonically in continuous search spaces, such as DE and PSO, are rarely adapted to suit combinatorial problems. For the RFID design scenario presented here, in which meander lines need to be constructed, a novel DE method has been developed (and refined). Indeed, the move to a continuous solution space enables the adaptive interpretation of solutions and promotes longer antenna designs [22]. By using a continuous to discrete mapping scheme based on encoding the relative direction of travel, an objective biasing mechanism, and a local search mechanism that regenerates real-valued solution vectors from modified antenna designs, the approach can produce antennas of the calibre of those from a well-established suite of ACO solvers while using fewer function evaluations. This success demonstrates that there are further applications that can reasonably start to be explored by continuous solvers (see also Hettenhausen, Lewis, Thiel and Shahpari [19]), and that the algorithmic improvements presented here may be useful additions, either singly or in combination. For example, future work can replace the multiobjective DE used here with other continuous solvers, such as multiobjective particle swarm optimization (MOPSO, see, e.g., [38]) to test the robustness of each of the proposed components. Another potentially rich area for future study is the use of a bias on the solution encoding to favour particular kinds of move, which recent work [31] has demonstrated can lead to the evolution of paths with “plough-like” shapes that the present algorithm appears less likely to produce (see Fig 8).

Given that different search spaces for the same problem produce different search space topologies, the ability for an algorithm to work in more than one will likely be a key to success on difficult problems into the future. The present findings demonstrate that the method of reintegrating discrete solutions produced by local search into a population of real-valued vectors should be considered carefully. Further investigation is warranted into the interaction between the mechanism used and the evolution of the population of real-valued solutions given the particular continuous evolutionary algorithm employed.
